# Splanchnic Vein Thrombosis: Etiology, Diagnosis, and Treatment

**DOI:** 10.1155/2015/506136

**Published:** 2015-10-25

**Authors:** Xingshun Qi, Valerio De Stefano, Marco Senzolo, Hao Xu, Andrea Mancuso

**Affiliations:** ^1^Department of Gastroenterology, General Hospital of Shenyang Military Area, No. 83 Wenhua Road, Shenyang 110840, China; ^2^Xijing Hospital of Digestive Diseases, Fourth Military Medical University, Xi'an 710032, China; ^3^Institute of Hematology, Catholic University, Largo Gemelli 8, 00168 Rome, Italy; ^4^Multivisceral Transplant Unit, Gastroenterology, Department of Surgical, Oncology and Gastroenterology, University Hospital of Padua, 35121 Padua, Italy; ^5^Department of Interventional Radiology, The Affiliated Hospital of Xuzhou Medical Colleges, Xuzhou 221006, China; ^6^Epatologia e Gastroenterologia, Ospedale Niguarda Cà Granda, Piazza Ospedale Maggiore 3, 20162 Milano, Italy; ^7^Medicina Interna 1, Azienda di Rilievo Nazionale ad Alta Specializzazione Civico-Di Cristina-Benfratelli, Piazzale Liotti 4, 90100 Palermo, Italy

This special issue was formally launched in May 31, 2014. Since then, all guest editors made lots of efforts for inviting and reviewing the submissions. Peer-reviewers also contributed their invaluable comments to the special issue. After about one year, a total of 9 high-quality papers were finally approved for the publication.

In the paper titled “Recurrent Thrombotic Events after Discontinuation of Vitamin K Antagonist Treatment for Splanchnic Vein Thrombosis: A Multicenter Retrospective Cohort Study” N. Riva et al. performed a multicenter retrospective cohort study to evaluate the risk of recurrent thrombotic events in SVT patients after discontinuation of anticoagulant treatment. Ninety patients were enrolled from 37 Italian anticoagulation clinics. All SVT patients had previously received anticoagulant treatment with warfarin. The overall incidence of recurrent thrombotic events was 3.3/100 patient-years. The results of two important subgroup analyses should be highlighted. First, the recurrence rate was 2.4/100 patient-years in patients with unprovoked SVT, 10.2/100 patient-years in those with permanent risk factor, and 0/100 patient-years in those with transient risk factor. On the basis of these impressive findings, long-term anticoagulation therapy should be worthwhile for SVT patients with permanent risk factor but not those with transient risk factor. Second, the recurrence rate was 19.1/100 and 2.0/100 patient-years in patients with and without liver cirrhosis, respectively. Accordingly, the usefulness of long-term anticoagulation for the prevention of thrombotic recurrence in liver cirrhosis may be emphasized. Certainly, the risk of bleeding caused by anticoagulation in such patients should be fully balanced.

In the paper titled “Imaging Diagnosis of Splanchnic Venous Thrombosis” S. Rajesh et al. provided a pictorial review to introduce the radiological manifestations of BCS, PVT, and mesenteric vein thrombosis. In their paper, there were up to 54 graphic illustrations or radiological images, which should be helpful for the physicians to improve the knowledge regarding the imaging diagnosis of SVT.

In the paper titled “Prevalence of Splanchnic Vein Thrombosis in Pancreatitis: A Systematic Review and Meta-Analysis of Observational Studies” W. Xu et al. systematically identified 44 papers to explore the prevalence of SVT in patients with pancreatitis. Overall, the pooled prevalence of SVT in pancreatitis was high (13.6%). Furthermore, the subgroup results were impressive. The prevalence was 16.6% in acute pancreatitis and 11.6% in chronic pancreatitis. The prevalence of splenic vein thrombosis was the highest (11.2%), followed by that of PVT (6.2%) and mesenteric vein thrombosis (2.7%). European patients had the highest prevalence of SVT (16.9%), followed by American (11.5%) and Asian (8.5%) patients.

In the paper titled “Systemic Venous Inflow to the Liver Allograft to Overcome Diffuse Splanchnic Venous Thrombosis” C. Lupascu et al. reported their surgical experiences to facilitate liver transplantation in the setting of diffuse SVT. Five patients with preoperatively confirmed extensive SVT successfully underwent cavoportal hemitransposition and renoportal anastomosis to ensure adequate allograft portal flow. However, the authors found that previous radiation-induced peritoneal injury should be excluded from the procedures. Additionally, the complications of (segmental) portal hypertension should be closely followed.

In the paper titled “Budd-Chiari Syndrome in China: A Systematic Analysis of Epidemiological Features Based on the Chinese Literature Survey” W. Zhang et al. systematically analyzed the Chinese literatures regarding the epidemiology of BCS in China. They identified 20191 Chinese BCS cases reported by the end of 2013. Henan, Shandong, Beijing, Jiangsu, and Anhui were the top 5 high-prevalence areas, accounting for 80% of Chinese BCS patients. Interestingly, 4 of them were located at the downstream areas of Yellow River. Whether or not regional characteristics are associated with the occurrence of BCS in China should be explored in future. More importantly, the incidence/prevalence of BCS in China might be higher than in the West (the incidence was estimated to be 0.88/million per year, and the prevalence was estimated to be 7.69/million).

In the paper titled “Prevalence of Budd-Chiari Syndrome during Pregnancy or Puerperium: A Systematic Review and Meta-Analysis” W. Ren et al. systematically identified 20 papers to explore the prevalence of pregnancy-related BCS. Overall, the pooled prevalence of pregnancy in BCS patients was 6.8%, ranging from 0% to 21.5%. As the data was restricted to female patients, the pooled prevalence was increased to 13.1%. According to the regions, Africa had the highest prevalence (10.6%), followed by Asia (7.1%) and Europe (5.0%).

In the paper titled “The Significance of Serum CA-125 Elevation in Chinese Patients with Primary Budd-Chiari Syndrome: A Multicenter Study” D. Cheng et al. performed a multicenter study to explore the clinical significance of serum CA-125 levels in Chinese patients with primary BCS. A total of 243 BCS patients were enrolled from 3 hospitals. CA-125 levels were significantly higher in BCS patients than in healthy controls. In BCS patients, higher CA-125 levels correlated with the presence of ascites, higher AST and ALT levels, decreased albumin levels, and higher Rotterdam scores. More notably, higher CA-125 levels were significantly associated with worse survival and asymptomatic survival rates after interventional treatment. In future, CA-125 levels before interventional treatment should be regarded as an important factor for determining the prognosis of BCS.

In the paper titled “Association between JAK2 rs4495487 Polymorphism and Risk of Budd-Chiari Syndrome in China” P. Zhang et al. conducted a large case-control study to analyze the relationship of JAK2 rs4495487 with the risk of BCS. Overall, genomic DNA was obtained from 300 BCS patients and 311 healthy controls. The frequency of the minor C allele was not significantly different between the whole BCS cases and controls (20.0% versus 18.7%; *P* = 0.57). Additional findings were obtained according to the status of JAK2 V617F mutation. Compared with healthy controls, the BCS cases with positive JAK2 V617F mutation had a significantly higher frequency of the minor C allele (42.9% versus 18.7%; *P* < 0.01), but the BCS cases with negative JAK2 V617F mutation had a similar frequency (19.4% versus 18.7%; *P* = 0.57). Notably, the JAK2 V617F mutation was rarely observed in Chinese BCS patients. Additionally, JAK2 rs4495487 was not associated with survival of BCS patients. Thus, the clinical utility of JAK2 rs4495487 might be limited.

In the paper titled “Pharmacologic Prophylaxis of Portal Venous System Thrombosis after Splenectomy: A Meta-Analysis” X. Qi et al. systematically identified 8 papers to explore the role of pharmacologic prophylaxis of portal venous system thrombosis (PVST) after splenectomy. Prophylactic drugs included anticoagulants, thrombolytics, and prostaglandin E1. Overall, the pharmacologic prophylaxis significantly decreased the incidence of PVST after splenectomy (odds ratio = 0.33, *P* < 0.00001). According to the indications for splenectomy, the subgroup analysis supported the efficacy of pharmacologic prophylaxis in patients treated with splenectomy for portal hypertension but not in those treated with splenectomy for hematological diseases. Notably, the risk of bleeding was not significantly influenced. However, their findings were limited by the quantity and quality of included studies.

